# Facial Nerve Neurolymphomatosis That Extends to Both the Brainstem and Extracranial Regions

**DOI:** 10.7759/cureus.44551

**Published:** 2023-09-01

**Authors:** Takeshi Kondoh, Kana Lee, Masashi Higashino, Takashi Mizowaki, Hirotomo Tanaka, Yoshiyuki Takaishi

**Affiliations:** 1 Neurosurgery, Shinsuma General Hospital, Kobe, JPN; 2 Otolaryngology, Shinsuma General Hospital, Kobe, JPN

**Keywords:** brainstem, perineural tumor, facial nerve palsy, central nervous system lymphoma, radiosurgery

## Abstract

A 73-year-old female developed right facial paralysis of House-Brackmann (H-B) grade III and was diagnosed with Bell's palsy. After three months of steroid therapy, she developed progressive hearing loss, and an MRI revealed a tumor in the right internal auditory canal. Within a few months, the right facial nerve palsy recurred, and the patient was treated with Gamma Knife radiosurgery. The tumor in the irradiated region disappeared, but new dysphagia was observed, and a right parotid gland tumor was detected for the first time. Tumors of the right parotid gland and the digastric muscle of the jaw were surgically resected, and a diagnosis of diffuse large B-cell lymphoma was made. The tumor had invaded the cranial nerves and brainstem region, and the patient did not wish to undergo further medical therapy. This was a case of malignant lymphoma that started as facial paralysis and invaded the brainstem, and testing for possible lymphoma at an early stage prior to radiotherapy was desirable.

## Introduction

Tumors located in the internal auditory canal are usually vestibular schwannomas, and when the tumor extends into the facial nerve canal, facial schwannoma is one of the possible diagnoses [1-3]. On the other hand, a rare type of tumor that is not a schwannoma is known to develop in cranial nerves along the nerve sheath and is called a perineural tumor on imaging [4,5]. Among them, neurolymphomatosis rarely develops in cranial nerves as a primary lesion without or with systemic lymphoma, making it difficult to diagnose at the time of presentation [6]. In this study, we experienced a case that was initially diagnosed as Bell's palsy and later as facial schwannoma. We performed Gamma Knife radiosurgery on the tumor, but the tumor rapidly grew extracranially and into the brainstem.

## Case presentation

**Figure 1:**
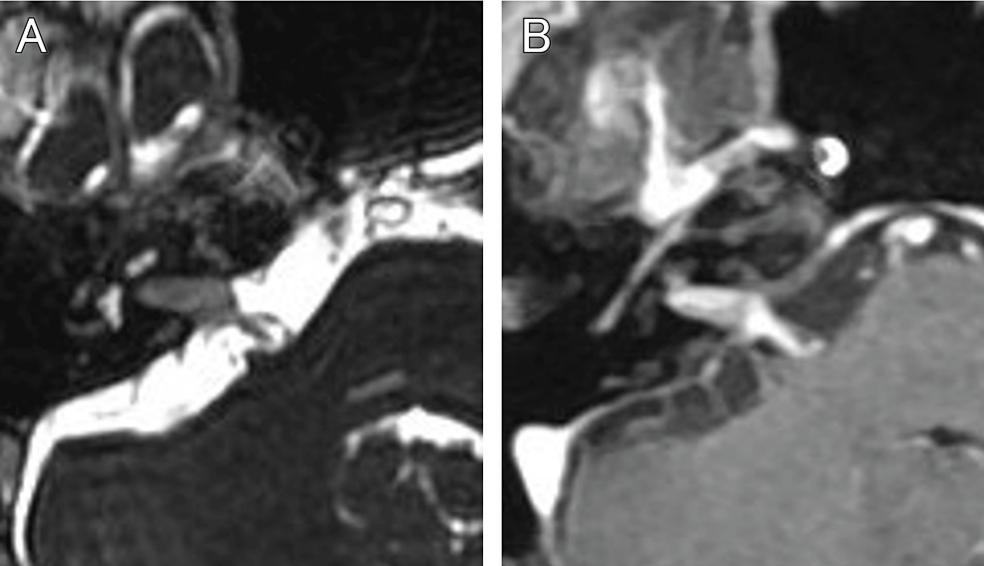
MRI at the initial diagnosis and at the treatment with Gamma Knife radiosurgery The initial T2-weighted image (A) and contrast-enhanced T1 image (B) show a tumor partially protruding from the internal auditory canal into the cisterna. MRI: magnetic resonance imaging

A 73-year-old female developed mild right facial paralysis, which was diagnosed as Bell's palsy by an otolaryngologist. She was treated with steroids, which resulted in the disappearance of her paralysis within a week. Three months after treatment for her facial paralysis, she presented with a chief complaint of progressive hearing loss on the right side. MRI revealed a tumor in the right internal auditory canal, and she was referred to our hospital with a diagnosis of acoustic neuroma. On the initial presentation, she was conscious and had lost effective hearing on the right side. MRI showed a tumor within her right internal auditory canal with a main diameter of 12 mm, associated with an enhanced lesion along the geniculate segment and the greater petrosal nerve (Figure 1A, 1B).

Given her very mild persistent facial nerve palsy, we considered the possibility of a facial schwannoma, and she decided to follow up on her neurological symptoms and imaging findings.

**Figure 2:**
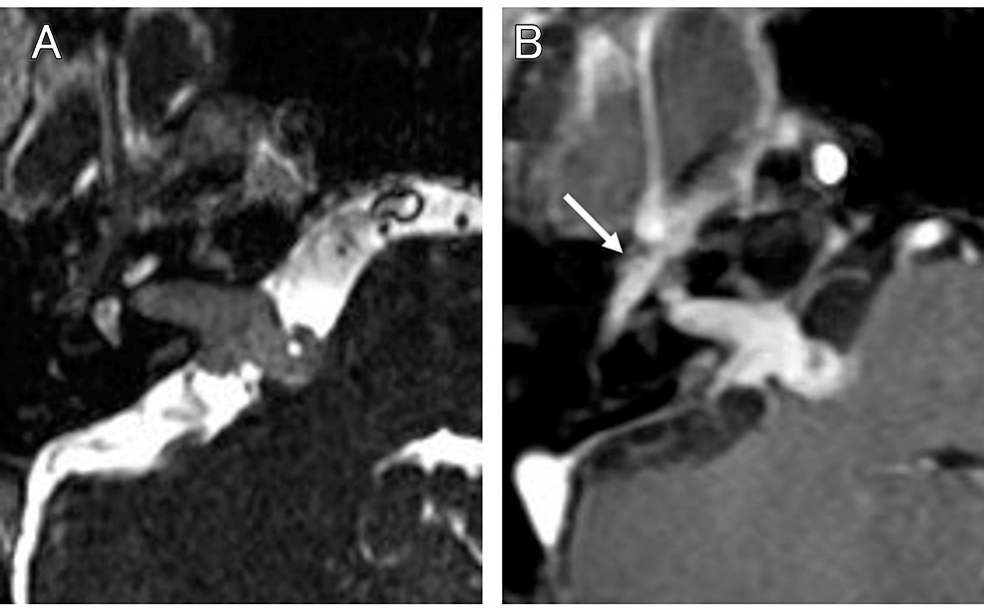
MRI three months after the initial diagnosis T2-weighted (A) and contrast-enhanced T1-weighted (B) images show that the tumor has grown and is in contact with the brainstem. The tumor in the internal auditory canal extends into the geniculate ganglion segment (arrow). MRI: magnetic resonance imaging

**Figure 3:**
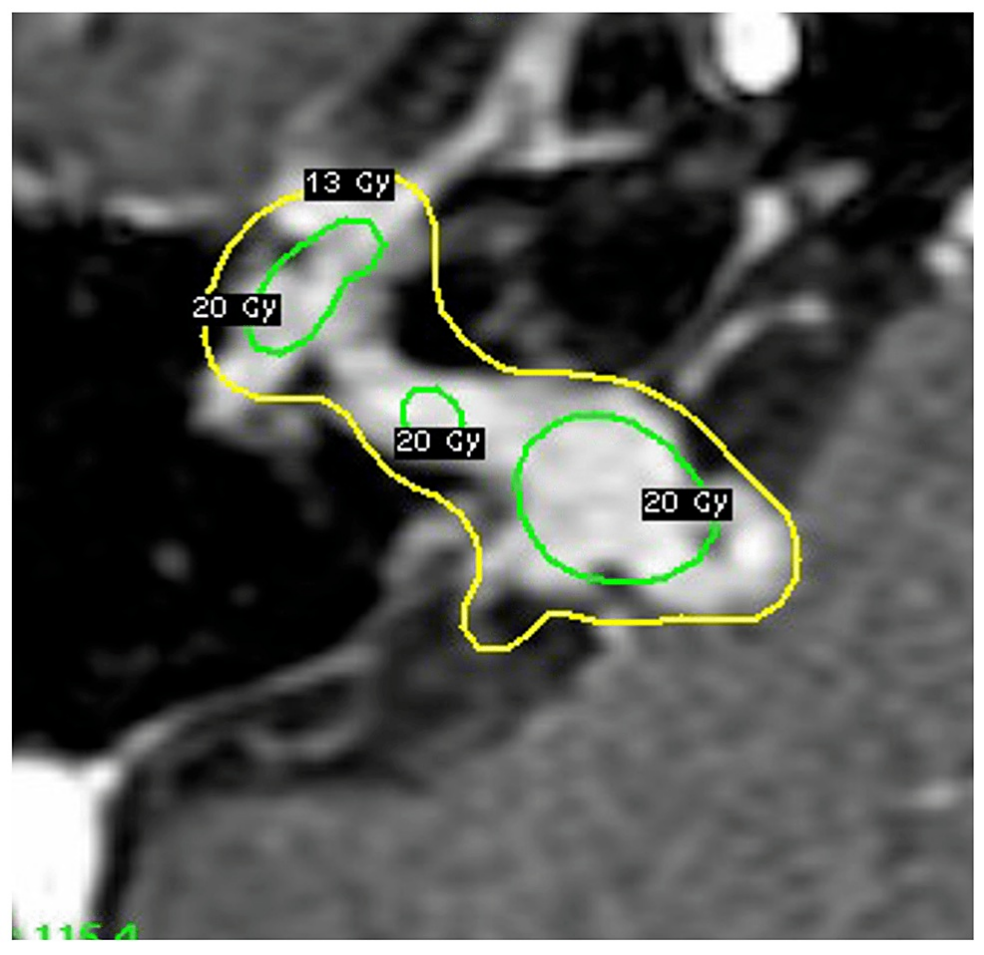
Gamma Knife planning and isodose line at 13 Gy (yellow) and 20 Gy (green) The tumor was treated with Gamma Knife therapy with a marginal dose of 13 Gy.

When she was examined three months later, her right facial palsy had progressed to the point where she had difficulty closing her right eye. She had nystagmus to the left, and her right hearing had completely disappeared. As a diagnosis other than facial schwannoma, the possibility of a malignant tumor or sarcoidosis was considered (Figure 2A, 2B), but as she did not wish to have a craniotomy to remove the tumor, which would also serve as a pathological diagnosis, Gamma Knife radiosurgery was first performed with 13 Gy at 50% marginal dose (Figure 3). The tumor volume was 1.6 cc.

**Figure 4:**
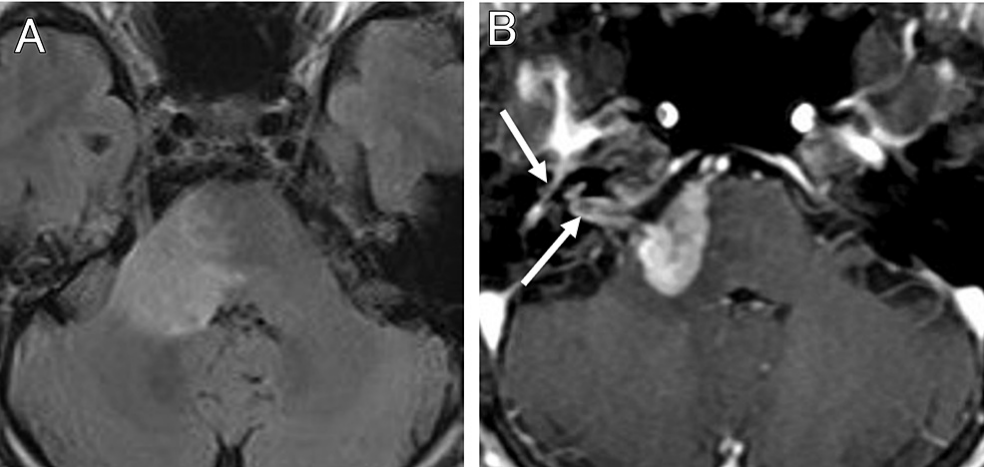
Follow-up MRI showing tumor invasion into the brainstem Two months after the treatment, FLAIR image (A), and contrast-enhanced T1-weighted image (B). The brainstem shows a mildly high-signal FLAIR image and a contrast-enhanced tumor on the right side. The tumor in the Gamma Knife-treated area has shrunk (arrows). MRI: magnetic resonance imaging, FLAIR: fluid-attenuated inversion recovery

**Figure 5:**
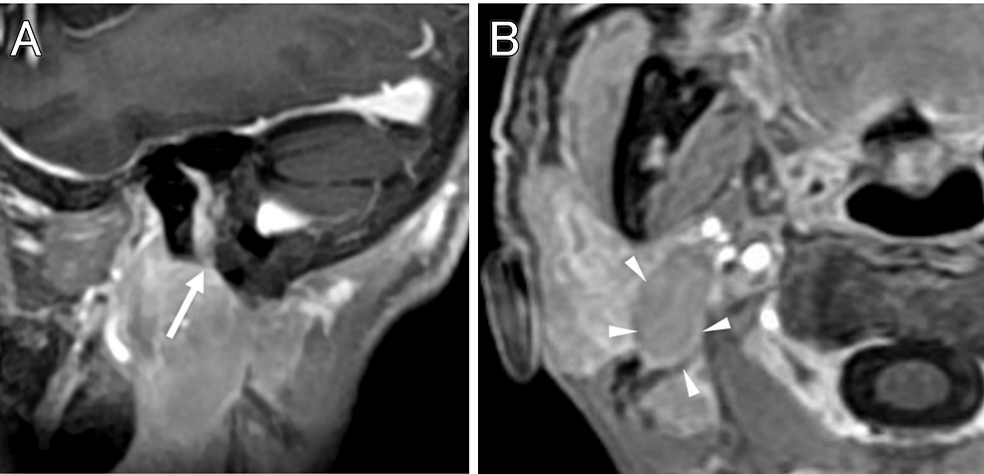
Follow-up MRI showing tumor extension to the parotid gland Contrast-enhanced T1-weighted image shows that the tumor has passed through the mastoid segment (C) (arrow) and reached the parotid gland (D) (arrowheads). MRI: magnetic resonance imaging

After two months of Gamma Knife treatment, dysphagia was observed. MRI revealed that the tumor invaded to the brainstem with diffuse edema (Figure 4A, 4B) and an extracranial tumor in the right parotid gland for the first time (Figure 5A, 5B). The tumor in the internal auditory canal disappeared, but a new contrast-enhancing lesion was found on the right side of the pons. Diffusion-weighted images showed mild hyperintensity in the brainstem lesion, but no such hyperintensity in the extracranial part.

CT of the chest and abdomen showed no neoplastic lesions. A cerebrospinal fluid (CSF) examination was performed and showed protein of 93 mg/dL, sugar of 54 mg/dL, cell count of 111/mm^3^, mononuclear cells of 105, polynuclear cells of 6, lactate dehydrogenase (LDH) of 40 IU/L, CEA (-), and interleukin (IL)-10 of 323 pg/mL. The cytology was class IIIa. The brainstem lesions continued to grow over a week, and a new mass lesion was found near the jugular foramen.

**Figure 6:**
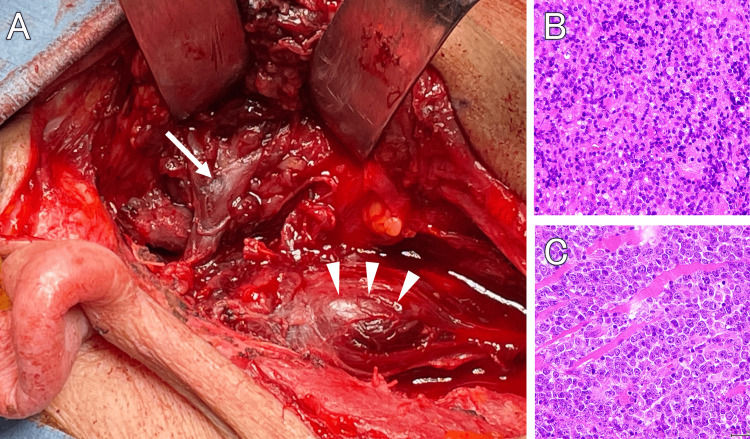
Intraoperative view and pathohistology of the tumor and postoperative MRI Intraoperative view (A) shows that the descending branches of the facial nerve (arrow) and the digastric muscle (arrowheads) are swollen with tumor. Histopathological study shows diffuse infiltration of tumor cells with large atypical cells that are poorly junctional (B,C). MRI: magnetic resonance imaging

While on steroids, the possibility of malignant lymphoma was considered, and tumors of the right parotid gland and digastric muscle were removed by an otolaryngologist (Figure 6A). Pathology showed a tumor with diffuse invasive growth in both the facial nerve (Figure 6B) and the digastric muscle (Figure 6C). The tumor was diagnosed as diffuse large B-cell lymphoma with the following pathological findings: atypical cells with poor conjugation and large karyoplasmic ratio, with irregular nuclei and anisotropic nuclei. The cytoplasm is barely visible, with a high Ki-67 labeling index of more than 90%. Expression of cell surface markers by flow cytometry revealed significant CD20 expression.

**Figure 7:**
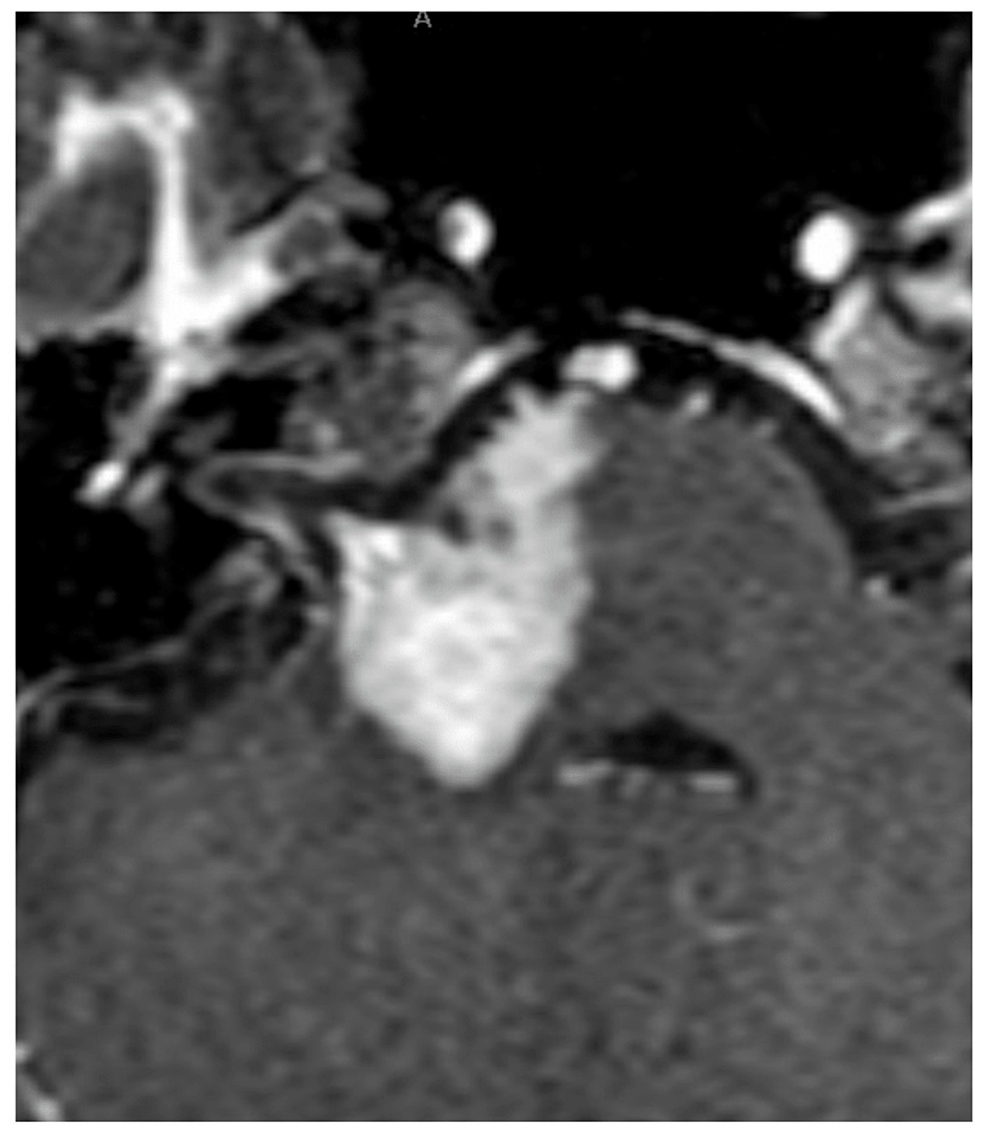
Contrast-enhanced T1-weighted MRI three months postoperatively MRI shows further enlargement of the tumor in the brainstem region, but no recurrence of tumor in the irradiated region. MRI: magnetic resonance imaging

Oral steroids temporarily improved the neurological symptoms. Contrast-enhanced T1-weighted images one month postoperatively show further enlargement of the tumor in the brainstem region (Figure 7). The patient did not want further treatment. She was discharged from the hospital and died two months later under palliative care.

## Discussion

Facial schwannoma is important in the field of neurosurgery as a tumor arising from the facial nerve, but its incidence is low at 1%-3% of cerebellopontine angle tumors and temporal bone tumors [3,7]. Differentiation from vestibular schwannoma is based on clinical symptoms such as dominant facial nerve palsy compared with hearing loss and imaging features such as the extension of the tumor from the internal auditory canal into the facial nerve canal. Surgery is sometimes performed, but in most cases, radiosurgery is preferred to preserve facial nerve function [1-3].

A diagnostic problem is differentiation from Bell's palsy [8,9]. Bell's palsy is thought to be the cause of 80% of hemifacial nerve palsies and, in many cases, has an acute onset, and symptoms improve with steroid administration, so routine imaging is not performed. It has been reported that MRI studies are performed in suspected cases of Bell's palsy with an atypical clinical course, resulting in the detection of undetected malignant tumors [10-12]. In the literature, there are only case reports, and the exact frequency of detection is not known. Many of these malignant tumors are carcinomas such as squamous cell carcinoma and adenocarcinoma. It is believed that the primary tumor in the neck can be found by performing extracranial MRI and contrast-enhanced examination with fat suppression.

In our case, Bell's palsy was initially diagnosed, and she was treated with steroids, which temporarily improved the facial nerve palsy. Although the cause of the onset is unknown because no MRI was performed, it is thought that lymphoma developed at this time. The first MRI, performed at the time of the hearing loss, showed an extension of the tumor in the facial nerve canal, and the main part of the tumor was in the internal auditory canal. Regarding the non-contrast MRI, the initial MRI did not show a clear extracranial space-occupying lesion, but the possibility of extracranial extension of the lymphoma was suspected. It should be noted that the extracranial lesion had a clear intraoperative border with the surrounding soft tissue and did not invade the surrounding area. In contrast, the disseminated tumor in the posterior fossa indicated that the tumor cells could easily spread within the skull.

It is well known in otolaryngology that malignant tumors arising from the parotid gland cause perineural invasion, and facial paralysis is the most common symptom [10,13]. Most of these are adenocarcinoma, squamous cell carcinoma, and salivary duct carcinoma, and lymphoma is extremely rare. On the other hand, neurolymphomatosis is a term used to describe lymphomas localized to nerves [6]. Neurolymphomatosis does not usually develop symptoms at the small single nerve such as the facial nerve [12]. The reported cases in the literature demonstrated the initial symptoms, and the primary site was mostly at the trigeminal nerve in the cranium [14-16]. In multiple spreading cases, it includes the spinal nerves [17-19]. Biopsies are often performed for definitive pathological diagnosis. It is well known that malignant lymphoma responds well to steroids in the early stages, and such a treatment may obscure the definitive diagnosis with imaging study. A diagnostic algorithm combining CSF diagnostic biomarkers, β2-microglobulin, soluble IL-2 receptor, interleukin-10, and C-X-C motif chemokine ligand 13, has been reported to have excellent diagnostic performance in terms of sensitivity and specificity, and biopsy can be avoided [20]. 

Also, in the present case, when the tumor extended to the brainstem, a CSF examination was performed, suggesting a high probability of lymphoma. Extracranial, but not intracranial, tumor excision was performed for pathological diagnosis.

We recommended aggressive treatment for her lymphoma, including chemotherapy, but she did not want further treatment because of severe dysphagia due to brainstem involvement. Early and appropriate lymphoma treatment could have been administered if an MRI scan had detected the tumor prior to steroid treatment for facial paralysis, along with a spinal fluid study. Since the number of reported cases is limited, the ordinal progression and deterioration patterns for neurolymphomatosis are hardly determined. The treatment of neurolymphomatosis includes chemotherapy consisting of rituximab, cyclophosphamide, doxorubicin hydrochloride, vincristine, and prednisolone (R-CHOP) or systemic methotrexate, in some cases followed by whole-brain radiation [15-17,19].

Considering the rarity of tumors that could mimic this condition, it is not typically practical to perform an MRI for every instance of facial palsy before initiating steroid therapy. In some cases, factors such as the patient's age or additional symptoms may influence the diagnostic approach. If the brainstem lesion had been detected before the dysphagia developed, re-irradiation with stereotactic radiotherapy could have been used to treat the brainstem lesion.

## Conclusions

Bell's palsy is a common cause of hemifacial paralysis and is not routinely assessed by MRI. Symptoms improve with steroid treatment. Our case demonstrated that such steroid treatment may lead to misdiagnosis of lymphoma and missed opportunities for appropriate treatment.
